# Supplementary material for: N6-Methyladenosine-Related lncRNA Signature Predicts the Overall Survival of Colorectal Cancer Patients
Source: Genes (Basel). 2021 Aug 31;12(9):1375. doi: 10.3390/genes12091375 (PMC8472391; doi:10.3390/genes12091375)
Supplement: Supplementary file 1 [file genes-12-01375-s001.zip › Supplementary Tables.pdf]

Supplementary Table S1 Summary of 23 recognized m6A regulators.

**m6A regulators**	**Type**
METTL3	writers
METTL14	writers
METTL16	writers
WTAP	writers
VIRMA	writers
ZC3H13	writers
RBM15	writers
RBM15B	writers
YTHDC1	readers
YTHDC2	readers
YTHDF1	readers
YTHDF2	readers
YTHDF3	readers
HNRNPC	readers
FMR1	readers
LRPPRC	readers
HNRNPA2B1	readers
IGFBP1	readers
IGFBP2	readers
IGFBP3	readers
RBMX	readers
FTO	erasers
ALKBH5	erasers

Supplementary Table S2 | The primer sequences for qRT-PCR.

**Gene**	**Primer Sequence**
AC003101.2	F: 5'-CTTGGGGGTGGGGAGAAC-3'
	R: 5'-GGGAGAGGAACAGGGGCT-3'
ITGB1-DT	F: 5'-GGAAACTTAGAAACCACC-3'
	R: 5'-GACAAAGAATCCAAAACA-3'
AC245041.1	F: 5'-AGTTTGAGAAGGCAGTGGCGTG-3'
	R: 5'-GGTGAGCTGAGGGTAAGGGGTG-3'
ZKSCAN2-DT	F: 5'-GCCAAATACGAGTAACCACAGC-3'
	R: 5'-GCCAACCTAAAACATAAAACCA-3'
AL391422.4	F: 5'-CACGACCAACTTTTATTC-3'
	R: 5'-TCTTGACACTCTCCATCT-3'
AC156455.1	F: 5'-CAGGAGCTAGAAATGTAG-3'
	R: 5'-GTAAGACCCAGTGAAAAC-3'
AC005229.4	F: 5'-AAACTAAAACACTACGCA-3'
	R: 5'-TAGAAGGATCACACAGAC-3'
GAPDH	F: 5'-GGAGTCCACTGGCGTCTTCA-3'
	R: 5'-GTCATGAGTCCTTCCACGATACC-3'

Supplementary Table S3 Univariate Cox regression analysis of lncRNAs associated with OS of CRC patients.

**LncRNAs**	**HR**	**95% CI**	**P value**
AC003101.2	2.837583	(1.474-5.461)	0.001
AP006621.2	1.21391	(1.086-1.357)	< 0.001
AC010463.3	1.829169	(1.188-2.816)	0.006
AL391684.1	2.043285	(1.234-3.385)	0.005
PRKAR1B-AS2	1.959419	(1.181-3.251)	0.009
ITGB1-DT	2.323974	(1.384-3.903)	0.001
AC104794.3	1.332002	(1.092-1.625)	0.004
AC133540.1	1.370284	(1.082-1.735)	0.008
AC145285.2	3.957493	(1.452-10.786)	0.007
AC016394.3	1.174474	(1.047-1.318)	0.006
MALINC1	3.108475	(1.379-7.008)	0.006
AP001619.1	1.618401	(1.161-2.256)	0.004
AL033384.2	1.674229	(1.146-2.447)	0.007
AL138756.1	1.228389	(1.059-1.425)	0.006
SEPTIN7-DT	4.759681	(1.83-12.36)	0.001
AC107375.1	1.303597	(1.068-1.591)	0.009
AC245041.1	1.409617	(1.160-1.714)	< 0.001
ZKSCAN2-DT	1.41382	(1.200-1.665)	< 0.001
AL391422.4	1.589563	(1.169-2.161)	0.003
AL161729.4	1.409218	(1.163-1.707)	< 0.001
AC016737.1	1.530942	(1.1164-2.099)	0.008
RPARP-AS1	1.238347	(1.066-1.439)	0.005
AL135999.1	1.817566	(1.161-2.847)	0.009
AC009404.1	1.948573	(1.193-3.181)	0.007
NSMCE1-DT	13.33001	(3.132-56.735)	< 0.001
AC009549.1	3.459762	(1.394-8.590)	0.007
AC013652.1	1.759801	(1.200-2.580)	0.003
AC156455.1	1.199453	(1.097-1.312)	< 0.001
AC005229.4	1.494943	(1.122-1.992)	0.006
