# Supplementary material for: N6-Methyladenosine-Related lncRNA Signature Predicts the Overall Survival of Colorectal Cancer Patients
Source: Genes (Basel). 2021 Aug 31;12(9):1375. doi: 10.3390/genes12091375 (PMC8472391; doi:10.3390/genes12091375)
Supplement: Supplementary file 1 [file genes-12-01375-s001.zip › Supplementary_Figures.pdf]

Supplementary Figures

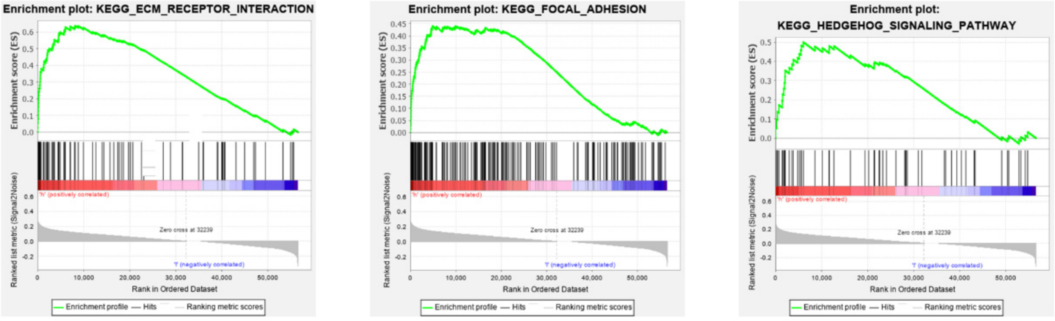

Supplementary Figure S1 Gene set enrichment analysis (GSEA) for risk score based on m6A-related lncRNAs.

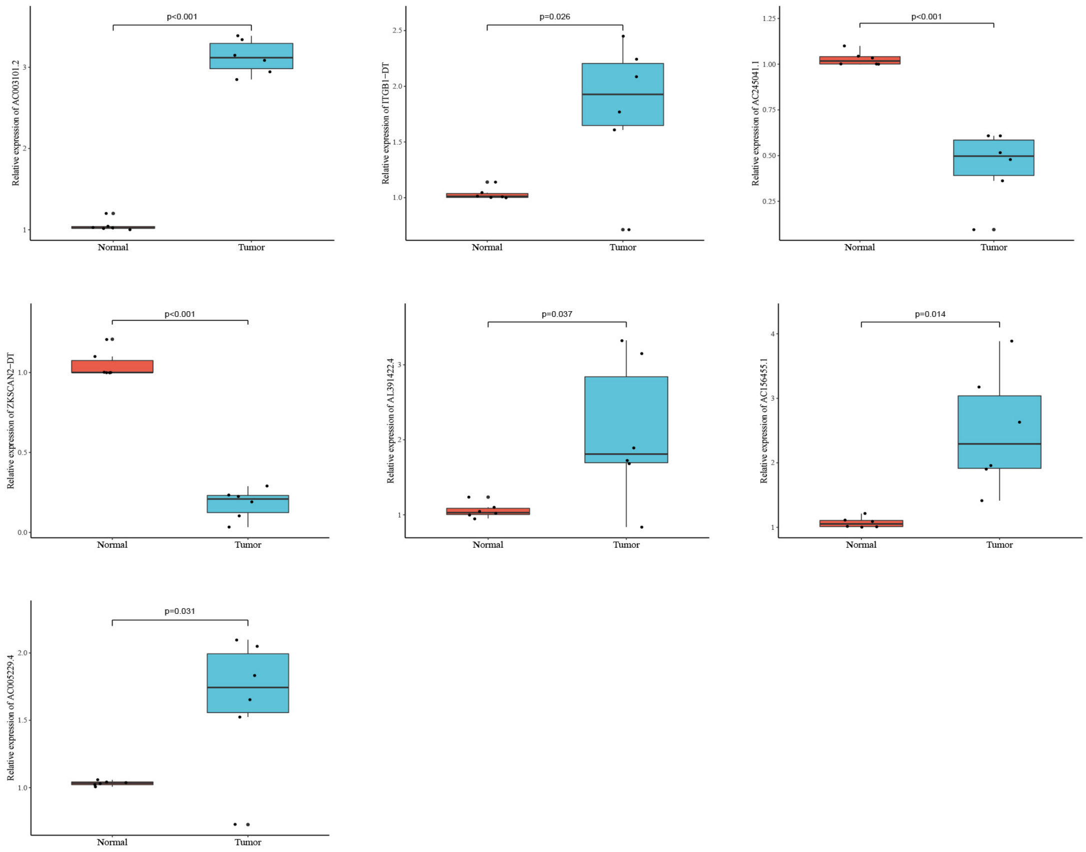

Supplementary Figure S2 Expression levels of 7 m6A-related lncRNAs of prognostic signature in colorectal cancer tissues and corresponding normal tissues by RT-PCR.
